# Numerical continuation of travelling waves and pulses in neural fields

**DOI:** 10.1186/1471-2202-14-S1-P70

**Published:** 2013-07-08

**Authors:** Hil GE Meijer, Stephen Coombes

**Affiliations:** 1 Department of Mathematics, University of Twente, Postbus 217, 7500 AE Enschede, The Netherlands; 2 Center for Mathematical Medicine & Biology, School of Mathematical Sciences, University of Nottingham, Nottingham, NG7 2RD, UK

## 

We study travelling waves and pulses in neural fields. Neural fields are a macroscopic description of the activity of brain tissue, which mathematically are formulated as integro-differential equations. While linear and weakly nonlinear analysis can describe instabilities and small amplitude patterns, numerical techniques are needed to study the nonlinear properties of travelling waves and pulses. Here we report on progress of such an approach using an excitatory neural field with linear adaptation. First, we find and analyse an anti-pulse, where the whole field is active except for a moving region with lowered activity. Such an anti-pulse may be relevant in modelling spreading depression. Second, we consider dynamics for relatively shallow, smooth activation functions where the neural field behaves as an excitable medium. We compute numerically the dispersion curves for travelling waves, i.e. the wavespeed as a function of the spatial period. This allows a kinematic analysis, see [3], that may be useful for analysing spreading epileptiform activity. Third, we present a numerical continuation method for periodic orbits of integro-differential equations based on fast Fourier transforms. Hence, neural fields with biophysically realistic mechanisms may be analysed beyond linearisation.

First we vary the strength of adaptation and observe that travelling fronts and pulses are organized by a heteroclinic cycle: a codimension 2 bifurcation. The unfolding uncovers a new type of solution that we call anti-pulse. This is a similar solution as in [2] but in a much simpler model. Following the construction as in [1] for pulses in the case of a Heaviside firing rate, we obtain equations for the width of the pulse and its speed. We solve these numerically as a function of the adaptation strength. Putting the asymptotics for the speed of (anti-)front and (anti)-pulse solutions together we show that these four curves meet according to the scenario of a heteroclinic cycle.

Next we show numerically how these solutions persist for smooth activation functions. We find travelling waves that approach travelling pulses for increasing size of the domain. By continuation of these pulses we recover the heteroclinic cycle making it a unifying organising center. Then we vary the wavespeed as a function of the adaptation strength. Here we find typical behavior of excitable media with oscillatory behaviour of the dispersion curves. At every oscillation, a new solution branch emerges resulting in complex dynamics and multistability of travelling waves.

Finally, we consider travelling waves in one dimension with Gaussian spatial connectivity on an infinite or periodic domain. For exponential connectivity functions the integro-differential equation can be converted to a partial differential equation using Fourier techniques. While this method does not work for Gaussian connectivity, it can be employed numerically. We consider a co-moving frame with an equidistant mesh and apply standard pseudo-arclength continuation to track periodic solutions varying a parameter. Our findings suggest a qualitative similarity of the dynamics for Gaussian connectivity compared to exponential connectivity.

## References

[B1] Bressloff PC Spatiotemporal dynamics of continuum neural fields Journal of Physics A 2012 45 0330012

[B2] Laing C Coombes S The importance of different timings of excitatory and inhibitory pathways in neural field models Network: Computation in Neural Systems 2006 17 151 1723 10.1080/09548980500533461 16818395

[B3] Keener J Sneyd J Mathematical Physiology 1998 Springer

